# Response to letter regarding “Puppyhood diet as a factor in the development of owner‐reported allergy/atopy skin signs in adult dogs in Finland”

**DOI:** 10.1111/jvim.16489

**Published:** 2022-07-21

**Authors:** Manal B. M. Hemida, Kristiina A. Vuori, Robin Moore, Johanna Anturaniemi, Sarah Rosendahl, Stella Maria Barrouin‐Melo, Anna Hielm‐Björkman

**Affiliations:** ^1^ Department of Equine and Small Animal Medicine, Faculty of Veterinary Medicine University of Helsinki Helsinki Finland; ^2^ Department of Nutrition and Clinical Nutrition, Faculty of Veterinary Medicine Beni‐Suef University Beni‐Suef Egypt; ^3^ Department of Veterinary Anatomy, Pathology and Clinics School of Veterinary Medicine and Zootechny, Federal University of Bahia Salvador Brazil

Dear Editor,

We read Drs McKenzie and Larsen letter with interest, and we thank the Editor for the opportunity to discuss the scientific questions through this forum.

Dr McKenzie (skeptvet^1^) and Larsen in their letter to the editor indicate that the DogRisk research group has a priori notions about the benefits of raw feeding. We want to point out that we only have three veterinarians and only three dog owners in our research group of eight, only two feeding raw, and that we take pride in discarding our hypotheses every time we prove them wrong. Regarding raw food we have time after time, through published articles and 10 university student theses in Finnish, seen that there are health benefits when owners use a raw diet for their dogs, compared to a dry. We acknowledge the variety of raw and dry dog diets available and therefore we also know that the reasons behind the health benefits are hard to estimate. We have analyzed gene‐expression^2^ and metabolomics^3^ in a raw‐dry diet‐intervention study. We look at processing, the absence of heat, macronutrient profile (proteins, fats, and carbohydrates, content and origin), bacterial load, among other factors. We now work with 10 hypotheses of why the raw food diet repeatedly comes up as healthier in our studies. In a world where our dogs suffer increasingly from noncommunicable diseases that are similar to those of humans (atopy, allergies, IBD, diabetes, certain cancers and similar) we can also make veterinary medicine relevant for human medicine by reporting what we see in dogs, that share most of the other environmental factors with us, their human owners. To this end, diet is an excellent variable to investigate, as dog owners tend to keep their dogs on the same diets for extended periods of time, sometimes a whole lifetime. Also, because of diets being so homogenous, owners do not tend to forget or misrepresent it, as they might do about their own diet. These are our motivators, not proving a preconceived hypothesis.

The second issue that Dr McKenzie and Larsen were concerned about was survey problems. All relevant concerns are mentioned in the limitations part of the article, as they themselves point out. In contrast to what was said in the letter, we want to point out that our research is, and has been in all our papers, completely transparent. We always mention the reasons for omitting cases: for example, robot answers, dogs not reported to eat enough to stay alive, too young controls as they still could develop the disease etc. As expected from an academic research group, our survey is validated,^4^ including the dependent variable of this paper (allergy/atopy). We are of the same opinion with the authors of the letter that owners misperceive especially body condition score; our data show that only 13% of owners have reported that their dogs are overweight (12%) or obese (1%). But we also know that they are good at perceiving the clinical signs their dogs have: itching, ear, eye or skin infections, anal gland problems, teeth and gum problems etc., as those are the reasons that they contact their veterinarians for appointments. The owners in our survey also had the possibility to tick other skin related disorders (eg, hot spots, demodicosis, seborrhea etc.) than allergy/atopy. And as they did not, there are not so many other diagnoses than allergy/atopy they could have had, as flea allergic dermatitis is not a common entity in Finland.

Further, the authors of the letter to the editor addressed an issue we again take pride in, analysis of the data. We totally disagree with the notion of “cherry picking” data and instead again want to highlight that by putting all results in either the main article or supplemental material for other researchers to see, we can best forward this area of research. Regarding selection bias, when using backward stepwise regression, the computer starts with all variables and excludes the ones with the lowest coefficient of determination. The result is a computer‐generated final model, including the significant variables. Only the machine chooses, not humans so no bias is even possible. It is common practice to discuss the significant results and trends while the pre‐analyses are put into the supplemental data (S1‐2). Figure 2A,B in our article show that there are more non‐atopic dogs than atopic dogs when the dogs have been eating more than 10% raw food and on the contrary, more atopic dogs, when they have been eating more than 80% of their diets as dry. Not all 10% intervals will show significance between the two diets as there will not be enough cases at all intervals and it is also normal that some of 20 intervals (here one; eating 60% dry) will be out of line, for the same reason. That it is so consistent, is remarkable.

At the start of our response, we mentioned some of our hypotheses for why raw diets come up as healthy, but that we lack an answer to the “why?”. The authors to the letter also had many “If‐why?” questions. At this time, we do not have the answers, but we hope that we will be able to answer them within a couple of years.

Another issue raised was funding bias. Also here, we have been completely transparent as can be seen in our long list of funders. We have been able to attract funding, for example, from state funding bodies, foundations, private people and companies by crowdfunding, raw food companies etc. and we are equally thankful to all. However, the big traditional dry feed companies have not been interested in funding our research, despite enquires. On our website, we disclose and thank all our sponsors (Moomin trolls and all) while letter author McKenzie instead has paid advertisements from Hills' and Mars, for example, featuring the same Royal Canin advertisement on atopy diets both as “sponsored content” and in his “education center,” on his Veterinary Practice News homepage.^5^ Dr JA Larsen discloses her close co‐operation with Mars, Hills' and Nestle' Purina in the Conflict of Interest section in all of her published articles.

Finally, we would like to point out that we have no “raw food agenda” but if we find that the “raw” is of value, we feel that we have an ethical obligation to the community to report it. We will therefore now recommend that people who feed dry food supplement the diet with at least 20% raw. If the health benefits come from beneficial bacteria, we will recommend that they should be added to the dry food, etc. Also, contrary to the letter authors' comments, we have and will continue to report on raw feeding not being dangerous for neither dogs nor family. In our risk‐analysis study, we found only three verified cases of food pathogen transmission from raw dog food to humans in 16 475 households feeding raw and in 98 353 pet years at risk.^6^ This study has been replicated with similar results.^7^ As a university‐based independent research group, we are driven by a concern for the growing disease load in our dogs. We hope our research will benefit both canine and human health and therefore we report everything we find, as we find it.
